# Outcomes of Chimeric Antigen Receptor (CAR) T-Cell Therapy in Patients with Large B-Cell Lymphoma (LBCL): A Single-Institution Experience

**DOI:** 10.3390/cancers15184671

**Published:** 2023-09-21

**Authors:** Aaron Trando, Anna Ter-Zakarian, Phillip Yeung, Aaron M. Goodman, Ayad Hamdan, Michael Hurley, Ah-Reum Jeong, Dimitrios Tzachanis

**Affiliations:** 1 School of Medicine, University of California San Diego, La Jolla, CA 92093, USA; atrando@health.ucsd.edu; 2 Department of Medicine, Division of Blood and Marrow Transplantation, University of California San Diego, La Jolla, CA 92093, USA; 3 Master of Advanced Studies (MAS) Program in Clinical Research, University of California San Diego, La Jolla, CA 92093, USA

**Keywords:** chimeric antigen receptor T-cell therapy, axicabtagene ciloleucel, tisagenlecleucel, large B-cell lymphoma, allogeneic hematopoietic stem cell transplant

## Abstract

**Simple Summary:**

Chimeric antigen receptor (CAR) T-cell therapies targeting CD19 have greatly improved the outcomes of many B-cell non-Hodgkin lymphomas, including relapsed/refractory large B-cell lymphoma (R/R LBCL). Nevertheless, a significant number of patients do not benefit. In this single-center retrospective study of patients with R/R LBCL, the outcomes of 66 patients are reported. We demonstrate that older and more frail patients are able to safely undergo CAR T-cell therapy but have significantly more infectious complications. We also demonstrate that ≥2 sites of extranodal involvement and ECOG at the time of CAR T-cell therapy are significant predictors of progression-free survival. Lastly, we demonstrate that the outcomes of patients who relapse after CAR T-cell therapy are poor.

**Abstract:**

Chimeric antigen receptor T-cell (CAR T-cell) therapy has revolutionized the treatment of relapsed/refractory (R/R) large B-cell lymphoma (LBCL). We describe the real-world baseline characteristics, efficacy, safety, and post-relapse outcomes of adult patients with R/R LBCL who received CAR T-cell therapy at the University of California San Diego. A total of 66 patients with LBCL were treated with tisagenlecleucel or axicabtagene ciloleucel. The median age was 59.5, and 21% were over 70 years old. Additionally, 20% of the patients had an Eastern Cooperative Oncology Group (ECOG) performance score of ≥2. Cytokine release syndrome incidence was 88%; immune effector cell-associated neurotoxicity syndrome incidence was 56%. All-grade infection occurred in 48% of patients and in 79% of patients > 70 years old. Complete response (CR) was achieved in 53% and partial response in 14%. Median progression-free survival (PFS) was 10.3 months; median overall survival (OS) was 28.4 months. Patients who relapsed post-CAR T-cell therapy had poor outcomes, with a median OS2 of 4.8 months. Upon multivariate analysis, both ECOG (HR 2.65, 95% CI: 1.30–5.41; *p* = 0.007) and ≥2 sites of extranodal involvement (HR 2.22, 95% CI: 1.15–4.31; *p* = 0.018) were significant predictors of PFS. Twenty-six patients were R/R to CAR T-cell therapy; six patients were in remission at the time of data cut off, one of whom received allogeneic transplant. Overall, older patients can safely undergo CAR T-cell therapy, despite the increased risk of all-grade infection. In our cohort, ECOG performance score and ≥2 sites of extranodal disease are significant predictors of PFS.

## 1. Introduction

Chimeric antigen receptor T-cell (CAR T-cell) therapy has demonstrated groundbreaking success in treating multiple hematologic malignancies.

CAR T-cell therapies are currently approved by the US Food and Drug Administration (FDA) for use in a number of diverse hematologic malignancies, from the very first approval of tisagenlecleucel (tisa-cel) for relapsed/refractory (R/R) acute lymphoblastic leukemia to there now being six approved CAR T-cell products [1,2]. These therapies have greatly improved the outcomes of many types of R/R B-cell non-Hodgkin lymphomas (B-cell NHLs), such as diffuse large B-cell lymphoma (DLBCL), follicular lymphoma, and mantle cell lymphoma (MCL). Data from the ZUMA-1 [3] phase 2 trial yielded an objective response rate (ORR) of 82% and a complete response rate (CRR) of 54% among 111 patients with R/R DLBCL who were treated with axicabtagene ciloleucel (axi-cel). Meanwhile, the phase 2 study JULIET [4] evaluated tisa-cel in adult R/R DLBCL and demonstrated an ORR of 52% and CRR of 40% among 93 patients. These results paved the way for the FDA approval of axi-cel and tisa-cel for R/R large B-cell lymphoma (LBCL), as well as, more recently, lisocabtagene maraleucel (liso-cel) for R/R LBCL and brexucabtagene autoleucel for R/R MCL [5].

CAR T-cell therapy has shown a clear clinical benefit for many patients with R/R LBCL. Nevertheless, there remain limitations of CAR T cells that have prevented their effective utilization in some patients. First, CAR T-cell products are associated with a potentially debilitating side effect profile, including the serious risks of cytokine release syndrome (CRS) and immune effector cell-associated neurotoxicity syndrome (ICANS) [6]. Secondly, while approximately 50% of patients with R/R LBCL achieve long-term survival to CAR T-cell products, the remaining 50% represent a large proportion of patients whose disease will eventually progress.

In fact, these latter patients who fail to respond to CAR T-cell treatment face especially poor outcomes. Di Blasi and colleagues [7] found that among 238 patients with R/R DLBCL who progressed/relapsed after CAR T-cell therapy, median progression-free survival (PFS) was only 2.8 months, and median overall survival (OS) was similarly dismal at 5.2 months. The study also revealed that the choice of subsequent treatment post-CAR T-cell therapy varied greatly, including lenalidomide, bispecific antibodies, targeted treatment, radiotherapy, and combined immunochemotherapy; disappointingly, the ORR to these therapies as an aggregate was 14.1%, as >70% of the patients presented progressive disease (PD) as their best response [7].

One promising salvage therapy is allogeneic hematopoietic stem cell transplant (HSCT), which is not represented in the cohort examined by Di Blasi et al. A study conducted by Fried and colleagues [8] demonstrated a 2-year OS of 45% and PFS of 31% in 39 patients with R/R DLBCL who received allogeneic HSCT after failing CAR T-cell therapy; these rates are markedly higher than those that have been seen with other salvage therapies. There are, however, numerous barriers that preclude pursuing this option after CAR T-cell therapy, most commonly, rapidly progressive disease.

It is, thus, imperative to identify biomarkers that may predict outcomes, including toxicity development and overall therapy performance, to better guide the application of CAR T-cell therapy. Currently, these biomarkers are not clearly defined. At the same time, no standardized treatment approaches exist for patients who progress/relapse after CAR T-cell therapy. We conducted a single-center retrospective study of LBCL patients who received CAR T-cell therapy to describe our real-world experience with the safety and efficacy of CAR T-cell therapy and the outcomes after relapse.

## 2. Materials and Methods

### 2.1. Study Population

Patients with R/R DLBCL who received either tisa-cel or axi-cel at the University of California of San Diego (UCSD) between January 2016 and June 2022 were included; the date of data cutoff was 15 April 2023. The study was approved by the UCSD Institutional Review Board and conducted in accordance with institutional guidelines and the principles of the Declaration of Helsinki.

### 2.2. Data Extraction

A comprehensive retrospective review of the electronic medical records was performed on eligible patients. Data on baseline characteristics, safety outcomes, and efficacy outcomes were abstracted. The diagnosis of R/R DLBCL in all patients was confirmed via review of pathology reports in all patients, aligning with the 2016 World Health Organization guidelines [9]. The double expressor and double-hit status were recorded at the time of initial diagnosis. Safety outcomes included incidences of CRS and NT. Cytopenias and infectious complications were also obtained and graded within the first 6 months of CAR T-cell infusion based on the Common Terminology Criteria for Adverse Events (CTCAE) v5.0 criteria [10]. Immune effector cell-associated hemophagocytic lymphohistiocytosis-like syndrome (IEC-HS) was graded based on the American Society for Transplantation and Cellular Therapy criteria [11]. Treatment responses were determined based on the Lugano 2014 criteria [12]. ORR was defined as patients who had either a complete (CR) or partial response (PR) as their best response. Post-relapse treatments were obtained for patients who relapsed or were refractory to CAR T-cell therapy.

### 2.3. Statistical Analysis

Descriptive statistics were utilized to summarize patient characteristics and other variables. Dichotomous and continuous variables were compared using chi-square test, *t*-test, and non-parametric tests appropriate for the distribution of the variables.

PFS was defined as the date of CAR T-cell infusion to time of progression, relapse, or death. OS was defined as the date of CAR T-cell infusion to time of death. OS2 was defined as the time of relapse from CAR T-cell therapy to death. Correlation of biologically relevant predictors was evaluated via correlation matrix. Survival probability of PFS was computed using Kaplan–Meier (KM) method.

For predictors of outcomes, univariate and multivariate regression models were used. Univariable Cox regression models were performed for high-risk prognostic factors. Multiple multivariable Cox regression model with backward selection method was used to select significant covariates at the 0.05 level. Similar steps were applied for logistic regression of responses (e.g., best ORR and CR). Tree-structure Cox regression model was also used to identify important nodes for subgroup and relevant prognostic cutpoints based on log-rank statistics. *p* < 0.05 was considered as statistically significant for predictors and cutoff points.

All statistical analyses were performed using R studio version 2023.03.1.3.

## 3. Result

### 3.1. Baseline Patient Characteristics

A total of 66 patients with R/R DLBCL were included. At the time of CAR T-cell infusion (day 0), the median age was 59.5 years (range, 23–81), 21% of patients were over the age of 70, and 67% were male. Eighty percent of patients presented with an ECOG between 0 and 1. Further, 27 patients (41%) had a low HCT-CI score of 0 or 1, 27 patients (61%) had an intermediate score of 2 or 3, and 12 patients (18%) had a high score of ≥4.

Prior to receiving CAR T-cell therapy, patients received a median of three treatments (range, 1–7). Three patients (5%) received CAR T-cell therapy as second-line therapy, while the remaining 63 patients (95%) received it as a third or greater line of therapy. Nineteen patients (29%) underwent prior autologous stem cell transplant, while one patient (2%) underwent prior allogeneic stem cell transplant before receiving CAR T-cell therapy.

Axi-cel was administered in 89% of patients, and tisa-cel was administered in 11% of patients. Thirty-seven patients (56%) received bridging therapy (BT) with steroids, chemotherapy, and/or radiation therapy.

At the time of initial diagnosis, 34 patients (56%) had DLBCL characterized as double-expressor lymphoma, while 17 patients (28%) had double-hit lymphoma. Most patients continued to show persistent evidence of disease on their pre-CAR T-cell PET scan, with 59 patients (89%) having a PET Deauville score of either 4 or 5. At day 0, 25 patients (38%) also had ≥2 sites of extranodal disease involvement. Two patients had central nervous system involvement (Table 1).

### 3.2. Safety Outcomes

The incidence of CRS among all 66 patients was 88%; all but 1 of these patients experienced grades 1–2 CRS (Table 2). The incidence of CRS and ICANS by CAR T-cell product type is described in Table A1. The incidence of CRS among the 52 patients ≤ 70 years old and the 14 patients > 70 years old was 88% and 86%, respectively. The incidence of CRS among 53 patients with an ECOG of 0–1 was 91%, while the corresponding value among 13 patients with an ECOG of 2–4 was 77%.

The incidence of ICANS was 56%, with 26% of patients experiencing grades 3–4 ICANS. The incidence of ICANS among the 52 patients ≤ 70 years old and the 14 patients > 70 years old was 52% and 71%, respectively, which were not significantly different (z = 1.31, *p* = 0.19, 95% CI: 0.10–0.49). The incidence of ICANS among 53 patients with an ECOG of 0–1 was 55%, while the corresponding value among 13 patients with an ECOG of 2–4 was 62%.

All patients also experienced cytopenia, including grade 3–4 anemia in 68% of patients, grade 3–4 neutropenia in 100% of patients, and grade 3–4 thrombocytopenia in 61% of patients. Among all 66 patients, a total of 41 infectious events occurred within six months of CAR T-cell infusion, including 8 upper respiratory infections (including 2 cases of respiratory syncytial virus), 6 cases of COVID-19, 5 urinary tract infections, 5 enterocolitis events (including 2 cases of clostridium difficile infection), 5 lung infections (including 1 case of pneumocystis jiroveci infection), 3 sepsis events, and 2 febrile neutropenia events. Grade 3–5 infectious events occurred in 18 of 66 patients (27%). Apart from these infectious events, 23 cases of cytomegalovirus (CMV) viremia were also observed among 57 patients who were tested for CMV. Additionally, one patient developed grade 5 IEC-HS secondary to CAR T-cell therapy.

### 3.3. Efficacy Outcomes

**Figure 1:**
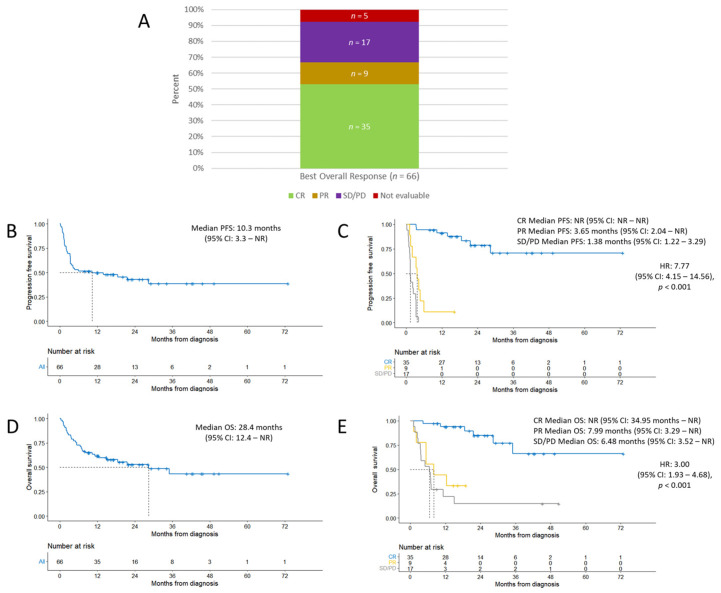
Efficacy outcomes. (**A**) Best overall response achieved. (**B**) Progression-free survival from date of CAR T-cell infusion to time of progression, relapse, or death. (**C**) Progression-free survival stratified by best overall response achieved. (**D**) Overall survival from date of CAR T-cell infusion to date of death. (**E**) Overall survival stratified by best overall response achieved. (**B**–**E**) Dashed lines represent median time. Abbreviations: CI, confidence interval; CR, complete response; HR, hazard ratio; NR, not reached; OS, overall survival; PD, progressive disease; PFS, progression-free survival; PR, partial response; SD, stable disease.

At a median follow-up time of 16.3 months, the ORR was 67%. CR was achieved in 35 of 66 patients (53%); 3 of the patients in CR have since relapsed. Among the remaining 31 patients, 9 patients (14%) achieved a PR, 17 patients (26%) had stable disease (SD) or PD, and 5 patients (8%) were unable to be evaluated (Figure 1A).

Median PFS was 10.3 months (95% CI: 3.3 not reached (NR)) (Figure 1B). Among patients who achieved CR, PR, or SD/PD, median PFS was NR (95% CI: NR–NR), 3.65 months (95% CI: 2.04–NR), and 1.38 months (95% CI: 1.22–3.29), respectively (Figure 1C), which were significant (HR 7.77, 95% CI: 4.15–14.56; *p* < 0.001).

The median OS in all patients was 28.4 months (95% CI: 12.4–NR) (Figure 1D). Among patients who achieved CR, PR, or SD/PD, median OS was NR (95% CI: 34.95–NR), 7.99 months (95% CI: 3.29–NR), and 6.48 months (95% CI: 3.52–NR), respectively (Figure 1E), which were also statistically significant (HR 3.00, 95% CI: 1.93–4.68; *p* < 0.001).

**Figure 2:**
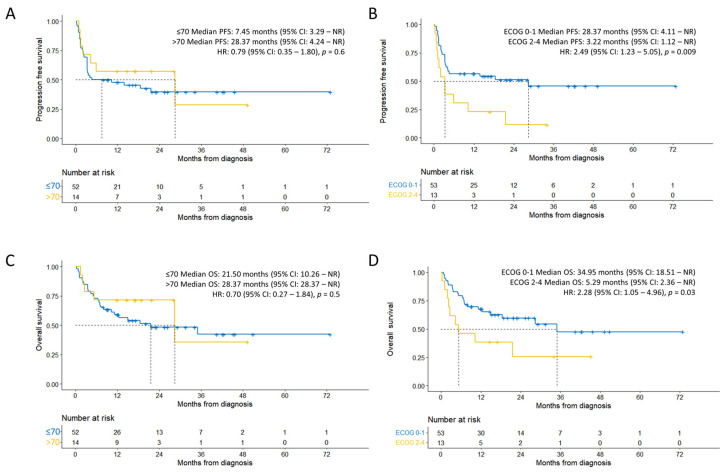
Efficacy outcomes sub-group analysis. (**A**) Progression-free survival stratified by age. (**B**) Progression-free survival stratified by ECOG status. (**C**) Overall survival stratified by age. (**D**) Overall survival stratified by ECOG status. (**A**–**D**) Dashed lines represent median time.

When stratified by age, the median PFS of individuals ≤ 70 years and > 70 years old was 7.45 months (95% CI: 3.29–NR) and 28.37 months (95% CI: 4.24–NR), respectively (Figure 2A), which were not significantly different (HR 0.79, 95% CI: 0.35–1.8; *p* = 0.6). When stratified by ECOG, the median PFS of individuals with an ECOG of 0–1 and 2–4 was 28.37 months (95% CI: 4.11–NR) and 3.22 months (95% CI: 1.12–NR), respectively (Figure 2B). Subjects with an ECOG of 0–1 had significantly longer PFS compared to subjects with an ECOG of 2–4 (HR 2.49, 95% CI: 1.23–5.05; *p* = 0.009).

When stratified by age, the median OS of individuals ≤ 70 years, and >70 years old was 21.50 months (95% CI: 10.26–NR) and 28.37 months (95% CI: 28.37–NR), respectively (Figure 2C), which were not significantly different (HR 0.70, 95% CI: 0.27–1.84; *p* = 0.5). When stratified by ECOG, individuals with an ECOG of 0–1 had significantly longer OS compared to those with an ECOG of 2–4 (HR 2.28, 95% CI: 1.05–4.96; *p* = 0.03), with a median OS of 34.95 months (95% CI: 18.51–NR) and 5.29 months (95% CI: 2.36–NR), respectively (Figure 2D).

CR was achieved in 27 of 52 patients ≤ 70 years old (52%); the corresponding CRR among 14 patients > 70 years old was 57%. CR was achieved in 30 of 53 patients (57%), with an ECOG of 0–1; the corresponding CRR among 13 patients with an ECOG of 2–4 was 38% (Table 3).

At the time of data cutoff, 31 of 66 patients (47%) had died. Twenty-three deaths were attributed to lymphoma progression. Four deaths were attributed to treatment-related mortality, which included cerebral bleed (*n* = 1), CRS (*n* = 1), neoplasm (*n* = 1), and multi-organ failure (*n* = 1) secondary to therapy. Three deaths were attributed to non-relapse mortality (NRM). Causes of NRM included multifocal pneumonia (*n* = 1), COVID-19 (*n* = 1), and rectal cancer (*n* = 1). The cause of death was unknown in one patient.

### 3.4. Factors Associated with Disease Relapse/Progression

Apart from a moderate correlation observed between age and age-adjusted HCT-CI (r = 0.489), low correlations (absolute r: 0.02 to 0.4) were observed between all baseline continuous variables (including age, age-adjusted HCT-CI, LDH, CRP, ferritin, extranodal involvement, and day 0 PET Deauville score). Univariate analyses of all patients’ (*n* = 66) best ORR and PFS utilizing logistic and Cox regression models, respectively, demonstrated extranodal sites ≥ 2 (OR 0.33, 95% CI: 0.10–1.06, *p* = 0.062; HR 2.10, 95% CI: 1.09–4.04, *p* = 0.027) and ECOG (OR 1.04, 95% CI: 0.26–5.25, *p* = 0.096; HR 2.49, 95% CI: 1.23–5.05, *p* = 0.012) as significant predictors (Table A2 and Table A3).

Based on backward selection criteria of *p*-value ≤ 0.2 for parameter selection, multivariate logistic regression for all patients (*n* = 66) for best ORR included double-hit status (yes or no), extranodal sites ≥ 2 (yes or no), ECOG status (0–1 and 2–4), and bridging (yes or no). No significant predictors were subsequently identified. Similarly, by applying the same parameter selection criteria, the multivariate Cox regression model (*n* = 66) included ECOG status (0–1 and 2–4), extranodal sites ≥ 2, double-expressor status, and day 0 PET Deauville score. Both ECOG (HR= 2.65, 95% CI: 1.30–5.41; *p* = 0.007) and extranodal sites ≥ 2 (HR 2.22, 95% CI: 1.15–4.31; *p* = 0.018) were identified as significant predictors of PFS (Table A2 and Table A3).

### 3.5. Outcomes after CAR T-Cell Therapy Relapse 

**Figure 3:**
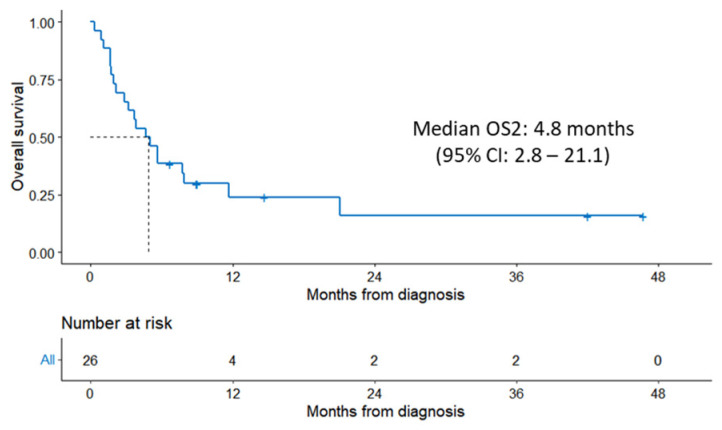
Kaplan–Meier estimate of OS2 from the date of relapse/progression after CAR T-cell therapy to the time of death. Dashed line represents median time.

Follow-up data were available for 26 patients who had relapsed/refractory disease to CAR T-cell therapy. The median OS of these 26 patients was 4.8 months (95% CI: 2.8–21.1) (Figure 3).

Patients received a median of one additional treatment after CAR T-cell therapy failure (range, 0–6). Twenty patients received a total of 40 additional treatment regimens after failing CAR T-cell therapy, while six patients did not receive any further treatment (Table 4). Six of twenty-six patients were alive at the time of last follow-up. One patient received allogeneic transplant, and five patients are responding to each of the following treatments: clinical trial with polatuzumab and mosunetzumab, bendamustine + rituximab + polatuzumab, tafasitamab + lenalidomide, loncastuximab, and venetoclax + rituximab.

Among the patients who had R/R disease after CAR-T therapy, 54% (14/26) were considered for treatment with allogeneic HSCT; nevertheless, only 1 patient underwent allogeneic HSCT, ultimately achieving a CR. The reasons for not proceeding to allogeneic transplant in the 25 patients were rapidly progressive disease (13 patients), age/comorbidities (4 patients), patient preference (3 patients), favorable response to salvage therapy (3 patients), lost to follow-up (1 patient), and post-operative complications (1 patient). The HCT-CI score post-relapse was available for 23 patients, and the score increased compared to pre-CAR T-cell therapy in 26% of the patients.

## 4. Discussion

CAR T-cell therapy has tremendously advanced the treatment of R/R B-cell lymphoma, with remarkable long-term survival rates of 40–50% [13,14,15]. The comparison of results from clinical trials to real-world outcomes provides useful insight for clinical management. Furthermore, given the inherent risk of adverse events from CAR T-cell therapy and the significant proportion of patients who do not benefit from CAR T-cell therapy, there is a great need to identify the predictive makers of efficacy. Therefore, we first aimed to describe the safety and efficacy outcomes of our institution’s patients with R/R LBCL who were treated with CAR T-cell therapy. We also aimed to identify the potential predictive factors of therapy response. Finally, we described the outcomes of non-responders.

The patient population in our study was older and frailer: 21% of patients were >70 years old and 20% had ECOG of ≥2, which is higher than what has been reported in clinical trials [13,14,15]. The percentage of patients with high-grade lymphoma with gene rearrangements in MYC and BCL2, BCL6, or both was similar. The use of BT has been of interest as a prognostic marker since patients with more aggressive disease may require bridging therapy. Studies have associated BT with mixed results, as one meta-analysis demonstrated that patients who received BT had a worse 1-year OS rate compared to those who did not; however, this discrepancy in outcomes may be explained by the heterogeneity in underlying disease status at baseline [16]. The trials that studied the three approved CAR T-cell products in second-line R/R LBCL each differed in their design with regard to incorporating BT. ZUMA-7 [15] allowed only corticosteroids, while BELINDA [13] and TRANFORM [14] allowed optional bridging chemotherapy. In our study, BT was administered in 56% of patients and did not predict ORR or longer PFS on multivariate analysis (Table A2 and Table A3).

Safety outcomes also appear to be comparable between our study and previously reported trials. CRS and ICANS in our cohort were managed per National Comprehensive Cancer Network (NCCN) guidelines [17]. The incidence of CRS and ICANS among our patient cohort was 88% and 56%, respectively. These outcomes align with the CRS incidence (92–93%) and ICANS incidence (60–64%) reported in the axi-cel trials [3,15]. It should be noted that 11% of the patients in our cohort received tisa-cel, which has been associated with lower rates of CRS (58–61%) and ICANS (10–21%) [4,13]. The rates of CRS and ICANS were similar between subjects > 70 years old and ≤70 years old. Among those with an ECOG of 0–1 versus 2–4, the incidence of CRS (91% and 77%, respectively) and ICANS (55% and 62%, respectively) was also comparable. In their matched control multicenter cohort study of 41 older patients (≥70 years old) and 41 younger patients (<70 years old) who received either tisa-cel or axi-cel, Ram and colleagues found no significant differences in the incidence of serious (≥grade 3) CRS and ICANS between elderly and younger patients [18]. Therefore, it appears that old and frail patients do not have an increased incidence of CRS or ICANS and, thus, it is safe for them to receive CAR T-cell therapy.

The incidence of serious infection (≥grade 3) in our cohort was 27%, while the corresponding rates were 14% in ZUMA-7 [15] and 20% in JULIET [4], the two trials for which data were available. Our incidence of serious infection likely appears higher than what has been observed in the clinical trial setting due to the small sample size of our cohort and older population. Those who were >70 years old had significantly higher rates of all-grade infection compared to those ≤70 years old; however, the PFS and OS between the two age groups were not significantly different. Notably, we did not determine if serious infections were treatment-related in this retrospective study. The incidence of NRM in our cohort (4.5%) was comparable with values that have been reported in other real-world datasets (4.4–6%) [19,20].

The incidence of CMV viremia specifically was not provided in any of the four major clinical trials. However, our incidence rate of 40% is consistent with another single center’s reported value of 44% [21]. Furthermore, 10 of the 23 patients diagnosed with CMV viremia required preemptive treatment with antiviral medications. Currently, the level of CMV viremia at which treatment is indicated remains unclear, and there was a propensity toward preemptive treatment during the early experiences with CAR T-cell therapy.

Our study showed an ORR of 67%, including a CRR of 53%. These values were comparable to the findings of both axi-cel trials, which demonstrated an ORR of 82–83% and CRR of 54–65% [3,15]. The CRR of patients ≤ 70 years and >70 years old was comparable at 52% and 57%, respectively; the CRR among patients with an ECOG of 0–1 and an ECOG of 2–4 was 57% and 38%, respectively. The response rates between axi-cel and tisa-cel were not compared due to the small sample size. One real-world comparison study of axi-cel and tisa-cel suggests that axi-cel may be more efficacious but carries greater risk of toxicity [22].

The median OS observed in our study was 28.4 months. Our study cohort included patients who were treated before and after the FDA approval of axi-cel in 2022 for patients with primary refractory disease or who relapsed within one year of treatment. Our results are comparable to the median OS of 25.8 months in ZUMA-1 [23]. The median overall survival in ZUMA-7 was not reached at a median follow-up of 47.2 months [24]. Meanwhile, our study’s median PFS was 10.3 months, which fell in the range of the PFS observed in the ZUMA trials (5.8–14.7 months) [3,14]. Akin to the landmark clinical trials, the most common cause of death in our study was also progressive disease. 

In our study, age stratification (≤70 vs. >70) did not result in a statistically significant difference in PFS or OS. In a retrospective study of 551 older patients (age ≥ 65) with DLBCL who received CAR T-cell therapy, Chihara and colleagues similarly found no significant difference in OS by age; however, patients age ≥ 75 had a significantly shorter 12-month event-free survival rate (34%) compared to patients aged 65–69 (43%) and aged 70–74 (52%) [25]. Our small sample size precluded analysis based on narrower age ranges. We did find stratification by baseline ECOG status (0–1 vs. 2–4) to be associated with a statistically significant difference in PFS and OS; the correlation between ECOG and PFS, in particular, was significant upon multivariate analysis.

There is currently no consensus on which biological, clinical, and/or imaging characteristics are most predictive of therapy outcomes. Among retrospective studies of patients with R/R LBCL who received axi-cel, one has linked low day-0 C-reactive protein and low peak ferritin with superior survival outcomes [26]. Another of these studies found day-30 SUVmax ≥ 10 on PET-CT scan to be a key predictive factor of disease progression in patients who achieved PR/SD [27]. In our univariate analysis, ≥2 extranodal sites prior to CAR T-cell therapy and a baseline ECOG of 2–4 predicted inferior ORR and shorter PFS; the involvement of ≥2 extranodal sites and an ECOG of 2–4 remained significant predictors of PFS on multivariate analysis. Notably, 10 of 23 (43%) patients who did not achieve CR at day 30 eventually achieved CR, stressing that the responses deepen post 30 days.

The outcomes after relapse from CAR T-cell therapy are extremely poor, as few effective salvage therapy options are available. Di Blasi and colleagues [7] reported a median OS2 of 5.2 months (95% CI: 4.1–6.6 months) in 238 patients who relapsed after CAR T-cell therapy. Similarly, our study yielded a comparable median OS2 of 4.8 months among 26 non-responding patients. Bispecific antibodies, such as glofitamab or epcoritamab, have recently shown promising results, yielding a CRR of 35% and 34.4%, respectively, among patients who had received prior CAR-T cell therapy [28,29]. These novel therapies were not available to our patients at the time of data collection in this study. 

The only curative treatment option in patients who fail CAR T-cell therapy at this time is allogenic transplant. Only one patient was able to proceed to allogeneic transplant (and currently remains in remission), while other patients received various salvage regimens. The principal reason prohibiting transplant was progressive disease. Also, the HCT-CI score increased in 26% of the patients who had relapsed when compared to pre-CAR-T cell therapy, suggesting that treatment-related toxicity could contribute to transplant ineligibility. One multicenter retrospective study found that the number of lines of therapy between CAR T-cell therapy and allogeneic HSCT along with disease status at time of allogeneic HSCT is predictive of response [8,30]. Further investigations of predictive markers are needed to appropriately select patients who may benefit from alternative approaches, including allogeneic transplant.

There are several limitations to this study. The single-institution nature of this study limited the sample size and statistical power. Also, the majority of the patients were treated with axi-cel; thus, comparative analyses were not conducted.

## 5. Conclusions

Here, we described the detailed characteristics and outcomes of patients with R/R DLBCL who received CAR T-cell therapy. In our patient series, which included old and frail patients, the response and toxicity rates were comparable to the results of currently published large-scale clinical trials in the literature. We also found ≥2 sites of extranodal disease involvement and ECOG to be predictive of PFS. Among non-responders to CAR-T cell therapy, progressive disease was the most common reason that prevented proceeding with allogeneic HSCT as salvage therapy. Thus, further research is needed to identify the potential predictive factors of response to CAR T-cell therapy, improving patient selection for CAR T-cell therapy versus other salvage therapy options.

## Figures and Tables

**Table 1:** Baseline characteristics of 66 R/R DLBCL patients receiving CAR-T therapy.

Characteristic	All
Number of patients	66
Age, years, *n* (%)	
≤70	52 (79)
>70	14 (21)
Median (range)	59.5 (23–81)
Male, *n* (%)	44 (67)
ECOG Score, *n* (%)	
0–1	53 (80)
2–4	13 (20)
Age-adjusted HCT-CI Score, *n* (%)	
Low: 0–1	27 (41)
Intermediate: 2–3	27 (41)
High: ≥4	12 (18)
Extranodal Involvement, *n* (%)	
<2	41 (62)
≥2	25 (38)
Genetics, *n* (%)	
Double Expressor	34 (52)
HGBCL with gene re-arrangements in MYC and BCL2, BCL6, or both	17 (26)
Missing Information	5 (8)
Pre-CAR T-cell Labs, median (range)	
LDH pre-LDT (*n* = 55) *	269 (121–1237)
LDH Day 0 (*n* = 66) *	265.5 (104–840)
CRP Day 0 (*n* = 45)	1.3 (0.05–18.43)
Ferritin Day 0 (*n* = 47)	1155 (20–5830)
Day 0 PET Deauville Score, *n* (%)	
1–3	6 (9)
4–5	59 (89)
Not evaluable	1 (2)
Bridging Therapy Type, *n* (%)	
None	29 (44)
Steroids	31 (47)
Chemotherapy	10 (15)
Radiation Therapy	2 (3)
Prior Lines of Therapy, median (range)	3 (1–7)
CAR T-cell 2nd Line of Therapy, *n* (%)	3 (5)
CAR T-cell ≥ 3rd Line of Therapy, *n* (%)	63 (95)
Prior Autologous Transplant, *n* (%)	19 (29)
Prior Allogeneic Transplant, *n* (%)	1 (2)
CAR T-cell Therapy Administered, *n* (%)	
Axicabtagene ciloleucel	59 (89)
Tisagenlecleucel	7 (11)

Abbreviations: CAR T-cell, chimeric antigen receptor T-cell; CRP, C-reactive protein; ECOG, eastern cooperative oncology group; HCT-CI, hematopoietic cell transplantation specific-comorbidity index; HGBCL, high grade B-cell lymphoma; LDH, lactate dehydrogenase; LDT, lymphodepleting therapy; PET, positron emission tomography; R/R DLBCL, relapsed/refractory diffuse large B-cell lymphoma. * Normal range for LDH was 25–175 units/liter.

**Table 2:** Safety outcomes after CAR T-cell therapy administration.

Adverse Event	All (%)	Grade 1–2 (%)	Grade 3–4 (%)	Grade 5 (%)
CRS (*n* = 66)	58 (88%)	57 (86%)	0 (0%)	1 (2%)
Age ≤ 70 (*n* = 52)	46 (88%)	45 (87%)	0 (0%)	1 (2%)
Age > 70 (*n* = 14)	12 (86%)	12 (86%)	0 (0%)	0 (0%)
ECOG 0–1 (*n* = 53)	48 (91%)	48 (91%)	0 (0%)	0 (0%)
ECOG 2–4 (*n* = 13)	10 (77%)	9 (69%)	0 (0%)	1 (8%)
ICANS (*n* = 66)	37 (56%)	20 (30%)	17 (26%)	0 (0%)
Age ≤ 70 (*n* = 52)	27 (52%)	14 (27%)	13 (25%)	0 (0%)
Age > 70 (*n* = 14)	10 (71%)	6 (43%)	4 (29%)	0 (0%)
ECOG 0–1 (*n* = 53)	29 (55%)	17 (32%)	12 (23%)	0 (0%)
ECOG 2–4 (*n* = 13)	8 (62%)	3 (23%)	5 (50%)	0 (0%)
Infection (*n* = 66) *	32 (48%)	16 (24%)	17 (26%)	1 (2%)
Age ≤ 70 (*n* = 52)	21 (40%)	12 (23%)	11 (21%)	0 (0%)
Age > 70 (*n* = 14)	11 (79%)	4 (29%)	6 (43%)	1 (7%)
ECOG 0–1 (*n* = 53)	23 (43%)	10 (19%)	13 (25%)	1 (2%)
ECOG 2–4 (*n* = 13)	9 (69%)	6 (46%)	4 (31%)	0 (0%)
CMV Viremia (*n* = 57)	23 (40%)	21 (37%)	2 (4%)	0 (0%)
Anemia (*n* = 66)	66 (100%)	21 (32%)	45 (68%)	0 (0%)
Neutropenia (*n* = 66)	66 (100%)	0 (0%)	66 (100%)	0 (0%)
Thrombocytopenia (*n* = 66)	66 (100%)	26 (39%)	40 (61%)	0 (0%)

Abbreviations: CMV, cytomegalovirus; CRS, cytokine release syndrome; ICANS, immune effector cell-associated neurotoxicity syndrome. * CMV viremia excluded.

**Table 3:** Efficacy outcomes (best overall response) stratified by age and ECOG status.

Characteristic	CR	PR	SD/PD	Not Evaluable
Age ≤ 70 (*n* = 52)	27 (52%)	5 (10%)	15 (29%)	5 (10%)
Age > 70 (*n* = 14)	8 (57%)	4 (29%)	2 (14%)	0 (0%)
ECOG 0–1 (*n* = 53)	30 (57%)	6 (11%)	14 (26%)	3 (6%)
ECOG 2–4 (*n* = 13)	5 (38%)	3 (23%)	3 (23%)	2 (15%)

**Table 4:** Treatment regimens administered post-CAR T-cell therapy progression/relapse.

Treatment Regimen	Number of Patients
Rituximab/obinatuzumab + bendamustine + polatuzumab	11
Lenalidomide + ibrutinib	1
Lenalidomide + tafasitamab	1
Loncastuximab	2
Pembrolizumab	1
Ibrutinib-based	1
Venetoclax-based	1
Chemoimmunotherapy, other	8
Radiation therapy	6
Clinical trial, cellular therapy	5
Clinical trial, other	2
Allogeneic hematopoietic stem cell transplant	1

## Data Availability

Research data that support the findings of this study are securely stored in an institutional repository and are available to share from the corresponding author upon reasonable request.

## Appendix A

**Table A1:** Safety outcomes after CAR T-cell administration by CAR T-cell product.

Adverse Event	All (%)	Grade 1–2 (%)	Grade 3–4 (%)	Grade 5 (%)
CRS (*n* = 66)	58 (88%)	57 (86%)	0 (0%)	1 (2%)
Axi-cel (*n* = 59)	52 (88%)	51 (86%)	0 (0%)	1 (2%)
Tisa-cel (*n* = 7)	6 (86%)	6 (86%)	0 (0%)	0 (0%)
ICANS (*n* = 66)	37 (56%)	20 (30%)	17 (26%)	0 (0%)
Axi-cel (*n* = 59)	34 (58%)	18 (31%)	16 (27%)	0 (0%)
Tisa-cel (*n* = 7)	3 (43%)	2 (29%)	1 (14%)	0 (0%)

Abbreviations: CRS, cytokine release syndrome; ICANS, immune effector cell-associated neurotoxicity syndrome; axi-cel, axicabtagene ciloleucel; tisa-cel, tisagenlecleucel.

**Table A2:** Univariate and multivariate logistic regression analysis to identify prognostic factors of ORR.

Variable	Univariate Analysis OR (95% CI) *p*-Value		Multivariate Analysis * OR (95% CI) *p*-Value	
Gender	0.73 (0.20–2.37)	0.61	—	—
Age	1.02 (0.98–1.07)	0.34	—	—
Age-adjusted HCT-CI Score	1.16 (0.82–1.73)	0.42	—	—
LDH Day 0	1.00 (1.00–1.00)	0.71	—	—
CRP Day 0	0.94 (0.80–1.08)	0.34	—	—
Ferritin Day 0	1.00 (1.00–1.00)	0.98	—	—
ECOG Score	1.04 (0.26–5.25)	0.096	0.28 (0.01–1.93)	0.85
Extranodal Involvement (≥2)	0.33 (0.10–1.06)	0.062	0.49 (0.13–1.76)	0.22
Double Expressor	0.50 (0.14–1.66)	0.26	—	—
HGBCL with gene rearrangements in MYC and BCL2, BCL6, or both	0.42 (0.12–1.54)	0.19	0.44 (0.11–1.69)	0.27
Bridging	0.42 (0.12–1.33)	0.14	0.62 (0.16–2.23)	0.34
Day 0 PET Deauville Score	0.82 (0.37–1.47)	0.53	—	—

Abbreviations: CI, confidence interval; CRP, C-reactive protein; ECOG, eastern cooperative oncology group; HCT-CI, hematopoietic cell transplantation specific-comorbidity index; HGBCL, high grade B-cell lymphoma; LDH, lactate dehydrogenase; OR, odds ratio; ORR, objective response rate; PET, positron emission tomography. * Variables for multivariate analysis were chosen based on backward selection criteria of a *p*-value ≤ 0.2 from univariate analysis.

**Table A3:** Univariate and multivariate Cox regression analysis to identify prognostic factors of PFS.

Variable	Univariate Analysis HR (95% CI) *p*-Value		Multivariate Analysis * HR (95% CI) *p*-Value	
Gender	1.22 (0.60–2.47)	0.60	—	—
Age	0.99 (0.97–1.01)	0.40	—	—
Age-adjusted HCT-CI Score	0.93 (0.76–1.15)	0.50	—	—
LDH Day 0	1.00 (1.00–1.00)	0.20	—	—
CRP Day 0	1.03 (0.96–1.11)	0.40	—	—
Ferritin Day 0	1.00 (1.00–1.00)	0.20	—	—
ECOG Score	2.49 (1.23–5.05)	0.012	2.84 (1.32–6.11)	0.008
Extranodal Involvement (≥2)	2.10 (1.09–4.04)	0.027	2.24 (1.05–4.77)	0.036
Double Expressor	1.72 (0.84–3.53)	0.14	1.65 (0.75–3.65)	0.20
HGBCL with gene rearrangements in MYC and BCL2, BCL6, or both	0.91 (0.41–2.00)	0.80	—	—
Bridging	0.88 (0.46–1.68)	0.70	—	—
Day 0 PET Deauville Score	1.64 (0.97–2.79)	0.066	1.13 (0.67–1.93)	0.60

Abbreviations: HR, hazard ratio; PFS, progression-free survival. * Variables for multivariate analysis were chosen based on backward selection criteria of a *p*-value ≤ 0.2 from univariate analysis.
